# The effect of brain serotonin deficit (TPH2-KO) on the expression and activity of liver cytochrome P450 enzymes in aging male Dark Agouti rats

**DOI:** 10.1007/s43440-023-00540-x

**Published:** 2023-10-17

**Authors:** Anna Haduch, Ewa Bromek, Wojciech Kuban, Agnieszka Basińska-Ziobroń, Przemysław J. Danek, Natalia Alenina, Michael Bader, Władysława A. Daniel

**Affiliations:** 1 Department of Pharmacokinetics and Drug Metabolism, Maj Institute of Pharmacology, Polish Academy of Sciences, Smętna 12, 31-343 Kraków, Poland; 2 Max-Delbrück-Center for Molecular Medicine, Berlin, Germany; 3 German Center for Cardiovascular Research (DZHK), Partner Site Berlin, Berlin, Germany; 4 Institute for Biology, University of Lübeck, Lübeck, Germany; 5 Charité University Medicine, Berlin, Germany

**Keywords:** Cytochrome P450 and b_5_, Rat liver, Ageing, Brain serotonin, TPH2-deficit, Serum hormones

## Abstract

**Background:**

Liver cytochrome P450 (CYP) greatly contributes to the metabolism of endogenous substances and drugs. Recent studies have demonstrated that CYP expression in the liver is controlled by the central nervous system via hormonal pathways. In particular, the expression of hepatic CYPs is negatively regulated by the brain serotoninergic system. The present study aimed to investigate changes in the function of the main liver drug-metabolizing CYP enzymes as a result of serotonin depletion in the brain of aging rats, caused by knockout of brain tryptophan hydroxylase gene (*TPH2-KO*).

**Methods:**

The hepatic CYP mRNA (qRT-PCR), protein level (Western blotting) and activity (HPLC), and serum hormone levels (ELISA) were measured in Dark Agouti wild-type (WT) male rats (mature 3.5-month-old and senescent 21-month-old) and in TPH2-KO senescent animals.

**Results:**

The expression/activity of the studied CYPs decreased with age in the liver of wild-type rats. The deprivation of serotonin in the brain of aging males decreased the mRNA level of most of the studied CYPs (CYP1A/2A/2B/3A), and lowered the protein level of CYP2C11 and CYP3A. In contrast, the activities of CYP2C11, CYP3A and CYP2C6 were increased. The expression of cytochrome b_5_ decreased in aging rats, but increased in TPH2-deficient senescent animals. The serum concentration of growth hormone declined in the aged and further dropped down in TPH2-deficient senescent rats.

**Conclusions:**

Rat liver cytochrome P450 functions deteriorate with age, which may impair drug metabolism. The *TPH2* knockout, which deprives brain serotonin, affects cytochrome P450 expression and activity differently in mature and senescent male rats.

**Supplementary Information:**

The online version contains supplementary material available at 10.1007/s43440-023-00540-x.

## Introduction

The human population of the contemporary world is aging rapidly. This phenomenon creates the need to understand the physiology of the aging body to be able to counteract adverse changes that reduce the quality of life in older people. With age, metabolic processes slowdown, which is manifested by significant changes in the functioning of most organs including the liver. Morbidity in aging people is significantly elevated, requiring the use of multiple drugs simultaneously. Seniors have an increasing risk of dangerous drug-drug interactions and are more vulnerable to adverse drug-drug effects than younger people [1, 2].

Liver cytochrome P450 (CYP) enzymes are important for the oxidative metabolism of endogenous substances and xenobiotics (drugs, carcinogens, and toxins). In general, aging is associated with a decline in the CYP enzymes’ activity. This decline can affect the metabolism and clearance of drugs and other substrates of CYPs. It was shown that the activities of human CYP1A2, CYP2D6, and CYP2E1 were lower in the liver microsomes of older people (60 + years) in comparison to young individuals (ca. 20 years) [3]. The same tendency was observed in the activity of CYP2B6 and CYP2C19, while the activity of CYP2A6 and CYP4A11 enzymes tended to increase with age [3]. The activity of CYP2C8/9 and CYP3A4 enzymes remained unchanged with increasing age [3], though the CYP3A protein level was elevated [4].

The results of animal studies also showed differences in hepatic cytochrome P450 expression and activity in different age groups. Yun et al. [5] demonstrated that the CYP1A1 protein was detected only in the liver of weanling rats and that the protein level of hepatic CYP1A2 and CYP2B1 declined in aging rats. It was also observed that the activities and protein levels of CYP2C11 and CYP3A2, the two main male-specific CYP enzymes, decreased with age [5–8]. Both enzymes remain under regulation by growth hormone (GH). The diminished GH level and disappearance of its pulsatile secretion can negatively affect the expression of CYP2C11 and CYP3A enzymes in senescent rats [8].

Changes in CYP enzyme activities that occur with age are not always consistent with alterations in their expression levels. In the case of liver CYP2E1, despite the increase in mRNA and protein levels of this enzyme, it has been reported that the CYP2E1 activity declines in senescent rats [9]. The reasons for the reduction in enzyme activity can be traced to oxidative stress which increases with aging and can probably cause posttranslational modification of CYP2E1 [6, 10]. In other studies, the mRNA level of CYP2D2 declined several-fold in older rats compared to newborn animals [11]. Moreover, studies of the metabolism of codeine and oxycodone, drugs specifically metabolized by CYP2D and CYP3A, suggested decreased activity of these enzymes in aging rats (18-month-old rats) [12].

In our earlier investigation of CYP2D in Dark Agouti rats, diminished protein levels and enzyme activity were found in the liver of senescent rats compared to the mature animals of both sexes [13]. Moreover, the deficiency of tryptophan hydroxylase 2 (TPH2), the main enzyme responsible for serotonin synthesis in the brain, significantly decreased the CYP2D protein levels and activity in the liver of male rats, while hepatic CYP2D of female rats was not altered, indicating sex-dependent dimorphism in the enzyme regulation in aging rats [13, 14]. However, other CYP enzymes were not investigated in this respect.

Our present work aimed to carry out complex studies into the expression and function of the main drug metabolizing CYP enzymes and cytochrome b_5_ in the liver of male rats (CYP3A, CYP2C11, CYP2C6, CYP2B, CYP2A, CYP1A) during aging and as a result of serotonin deprivation in the brain. Since our previous studies indicated that the brain serotonergic system affects the activity and expression of hepatic cytochrome P450 via neuroendocrine pathways (reviewed in [15]), it seemed highly advisable to investigate the effect of brain serotonin deficiency on hepatic CYP enzymes in aging animals, in which the classical way of serotonin synthesis in the brain was turned off by *TPH2* gene knockout. For this purpose, we performed comparative studies on mature, senescent, and senescent TPH2-KO rats, the latter as an experimental model of serotonin deficiency in the elderly brain [13].

## Materials and methods

### Chemicals

Nicotinamide adenine dinucleotide phosphate (NADP) and its reduced form (NADPH), glucose-6-phosphate-dehydrogenase, glucose-6-phosphate, caffeine and its metabolites and RNA-free water were obtained from Sigma (St. Louis, MO, USA). Testosterone and its hydroxylated metabolites were purchased from Steraloids (Newport, KY, USA). Warfarin and all the organic solvents were donated by Merck (Darmstadt, Germany), while 7-hydroxywarfarin was synthesized at Maj Institute of Pharmacology PAS (Kraków, Poland). The polyclonal primary anti-rat CYP1A1 antibody was from Millipore (Temecula, CA, USA). The polyclonal primary rabbit anti-rat CYP2C11 was obtained from Invitrogen Carlsbad, CA, USA; product number: PA3-034). The polyclonal primary rabbit anti-human CYP2A6 and CYP3A4 were from Fine Test (Wuhan, China; product numbers: FNab02157; FNab02165, respectively), the monoclonal mouse anti-rat CYP2B1/2 and CYP2C6 antibodies were from Santa Cruz Biotechnology (Dallas, TX, USA; product numbers: SC-53242; SC-73484, respectively). The polyclonal anti-mouse β-actin antibody was from Sigma Aldrich (St. Louis, Il, USA; product number: A1978). Horseradish peroxidase-labelled secondary antibodies: goat anti-mouse or goat anti-rabbit were from Vector Laboratories (Burlingame, CA, USA; product number: PI-1000) or Jackson ImmunoResearch (West Grove, PA, USA; product number: 115-035-003), respectively. Rat cDNA-expressed CYP enzymes were from Gentest Corp. (Woburn, MA, USA). The chemiluminescence reagents SuperSignal™ West Pico PLUS were provided by Thermo Scientific (Rockford, IL, USA; product number: 34077). Total RNA Mini kit from A&A Biotechnology (Gdynia, Poland; product number: 031-100) was used for RNA isolation. For mRNA estimation, a High-Capacity cDNA Reverse Transcription Kit, from Applied Biosystems (Waltham, MA, USA; product number: 4368814), TaqMan assays, and the TaqMan Gene Expression Master Mix from Life Technologies (Carlsbad, CA, USA; product number: 4369016) were used. The ELISA kits for measuring growth hormone and corticosterone came from FineTest (Wuhan, China; product number: ER0859).

### Animals

The in vivo experiments with rats were carried out according to the rules of the 86/609 EEC Directive and were accepted by the ethical committee of the local government (LAGeSo, Berlin, Germany), and the Local Bioethics Commission at the Institute of Pharmacology, Polish Academy of Sciences (Kraków, Poland). The animals were kept under standard conditions, maintaining a 12:12 h light/dark cycle, a room temperature of 22 ± 2 ºC, and a humidity of 55 ± 5%. The animals had free access to granulated food (Ssniff, Soest, Germany) and water.

Wild-type (WT) and TPH2-deprived (TPH2-KO) male rats of the Dark Agouti background originally generated by Kaplan et al. [16] were bred at the Max-Delbrück-Center for Molecular Medicine (Berlin, Germany) as reported earlier [13]. Comparative studies were conducted on three different animal groups: mature WT (3.5-month-old) and senescent WT Dark Agouti male rats, and senescent TPH2-KO Dark Agouti male rats (21-month-old) as a continuation of our previous studies on the CYP2D expression and function [13]. The overall number of animals was 50. One sample of blood (for serum hormone concentrations) and two samples of the liver (one for mRNA measurement and one for microsome preparation to measure CYP protein level and activity) were taken from each animal. All samples were taken for biochemical analysis.

### Determination of cytochrome P450 activity in liver microsomes

The animals were decapitated after 18 h of food deprivation. Whole livers derived from mature 3.5-month-old (TPH2-WT) or from senescent 21-month-old (TPH2-WT or TPH2-KO) Dark Agouti male rats were collected and stored at − 80 °C. Liver microsomes were isolated by the differential centrifugation method routinely used in our laboratory [17].

The activities of cytochrome P450 enzymes were assessed in liver microsomes by measuring the rates of specific metabolic substrates and reactions under linear conditions, as reported previously. In brief, reactions were carried out in an incubation mixture containing rat liver microsomes (ca. 1 mg of protein/ml), a NADPH or NADPH-generating system and a CYP-specific substrate. The activity of CYP1A was determined by measuring the rate of caffeine metabolism (100 μM), i.e., C-8-hydroxylation and 3-N-demethylation for 50 min. The metabolites formed from caffeine were analyzed by HPLC with UV detection [18]. The CYP2C6 activity was studied by estimating the rate of warfarin (60 μM) 7-hydroxylation proceeding for 15 min. The formation of warfarin 7-hydroxy-metabolite was monitored by HPLC with fluorescence detection [19]. The activities of other drug-metabolizing CYP enzymes (CYP2A, 2B, 2C11, 3A) were studied by measuring the rates of CYP-specific reactions: the 7α-, 16β-, 2α- and 16α-, and 6β-hydroxylation of testosterone, respectively. The substrate concentration was 100 μM and incubation time 15 min. The metabolites formed in the mentioned positions of testosterone structure were quantified by HPLC with UV detection [20].

### Measurement of CYP enzyme proteins in the rat liver microsomes

The CYP enzyme proteins in the liver of Dark Agouti male rats (5–10 µg of microsomal proteins) were estimated by Western blotting [21, 22]. Monoclonal anti-rat CYP2B1/2 and CYP2C6 antibodies (dilution 1:1000 and 1:2000, respectively), polyclonal anti-rat CYP1A1/2 and CYP2C11 antibodies (dilution 1:1000), monoclonal anti-human CYP2A6 (recognizing rat CYP2A) and CYP3A4 (recognizing rat CYP3A1/2) antibodies (dilution 1:1000 and 1:2000, respectively), and a secondary antibody (a species-specific horseradish peroxidase-conjugated anti-IgG) were applied (dilution 1:2000). Rat cDNA-expressed CYP enzymes were used as respective standards: CYP1A1, CYP1A2, CYP2A1, CYP2B1, CYP2C6 (5 µg), CYP2C11, CYP3A1, CYP3A2 (1 µg). The intensity y of the CYP protein bands was measured with the Luminescent Image Analyzer LAS-1000 (Fuji Film, Tokyo, Japan), and normalized to protein loading using β-actin (primary antibody dilution 1:10,000) [21, 22].

### Measurement of CYP mRNAs in the liver

#### Total RNA isolation and reverse transcription

The total RNA extraction from each sample was performed utilizing approximately 50 mg of rat liver tissue (Total RNA Mini Plus kit, A&A Biotechnology, Gdynia, Poland). For reverse transcription, approximately 2 µg of total RNA from each sample of liver tissue was converted to cDNA following the manufacturer protocol (High-Capacity cDNA Reverse Transcriptase kit, Applied Biosystems, Waltham, MA, USA).

#### Quantitative reverse transcription-polymerase chain reaction (TaqMan Analysis)

The cDNAs of genes coding for CYP enzymes (*CYP1A1, 1A2, 2A1, 2A2, 2B1, 2B2, 2C6, 2C11, 3A1, 3A2)* and cytochrome *b*_*5*_ were analyzed in rat livers using gene-specific TaqMan respective probe sets (Rn00487218_m1, Rn1422531_m1, Rn00562200_m1, Rn00582207_m1, Rn01457875_m1, Rn02786833_m1, Rn03417171_gH, Rn00569868_m1, Rn01412959_g1, Rn01412889_mH, Rn00573797_m1, respectively, from Applied Biosystems (Waltham, MA, USA). The use of specific probes designed for the β-actin gene (*Actb*; Rn00667869) were employed to serve as an internal reference to assess the expression of the *CYP* or *b*_*5*_ genes. The process involved conducting amplification on the QuantStudio 12 k Flex real-time polymerase chain reaction system (Thermo Fisher Scientific Inc., Waltham, MA, USA). The amplification was carried out over 40 cycles using standard conditions suitable for TaqMan-based assays in relative quantification mode. The QuantStudio 12k Flex system software was responsible for determining the threshold cycle (CT) values for the *CYP*, *b*_*5*_, and *Actb* genes. To evaluate the expression of the individual *CYP* and *b5* genes in relation to *Actb* expression, the comparative ΔCt method was applied. The relative expression of *CYP* and *b*_*5*_ genes was estimated by comparing their threshold cycle values to that of *Actb*. The relative-fold mRNA content was calculated using the formula 2^−ΔΔCt^.

### Analysis of hormone levels in the blood serum

The concentrations of growth hormone and corticosterone in blood serum were estimated using ELISA kits following the manufacturer’s instructions. A Synergy/HTX Multi-Mode Microplate Reader (BioTek, Winooski, VT, USA) was used to measure the intensity of absorbance.

### Data analysis

Alterations in the mRNA levels of *CYPs* and *b*_5_, and CYP protein levels and activities in senescent (senescent vs. mature WT) and TPH2-KO (senescent TPH2-KO vs. senescent WT) male rats were evaluated statistically. Statistical analysis was performed using GraphPad Prism 9 software (GraphPad Software, Inc., La Jolla, CA). Shapiro–Wilk tests were used to assess normality. For normally distributed groups, parametric unpaired Student’s t-tests were applied, while non-normally distributed groups were analysed using the nonparametric Mann–Whitney U test (MWU). The results were indicated as the mean ± SEM, and were recognized as significant when p ≤ 0.05.

## Results

### The influence of aging on the expression and activity of CYP enzymes in the liver of male Dark Agouti rats (senescent vs. mature WT rats)

In the first phase of our research, we conducted an introductory study of the expression and function of CYP enzymes in the liver of mature and senescent Dark Agouti male rats. The control (mature male rats, 3.5-month-old) and senescent (21-month-old) group contained 14 or 12 rats, respectively.

**Fig. 1:**
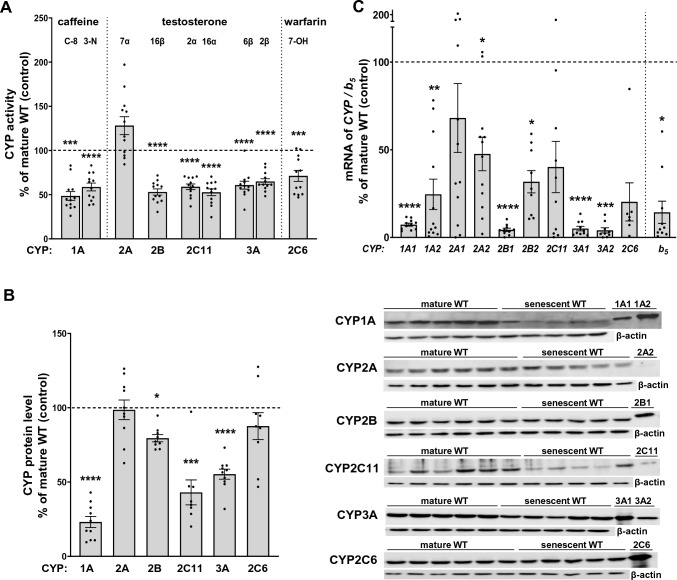
The activities of major cytochrome P450 enzymes and the expression levels of cytochrome P450 (CYP) and b_5_ genes in the liver of wild-type mature (mature WT) and wild-type senescent (senescent WT) Dark Agouti male rats. **A** The effect of age on CYP enzyme activities in the liver of senescent Dark Agouti wild-type rats (senescent WT). All values are the mean ± SEM of 12–14 rats. Statistical significance is shown as ***p < 0.001; ****p < 0.0001 vs. mature WT (Student’s t-test). The control values (nmol/mg of protein/min) are as follows: 0.0348 ± 0.0033 (CYP1A, caffeine C-8-hydroxylation); 0.0081 ± 0.0004 (CYP1A, caffeine 3-N-demethylation); 0.3521 ± 0.0392 (CYP2A, testosterone 7-α-hydroxylation); 0.0558 ± 0.0028 (CYP2B, testosterone 16-β-hydroxylation); 0.0057 ± 0.0002 (CYP2C6, warfarin 7-hydroxylation); 1.9076 ± 0.1012 (2C11, testosterone 2-α-hydroxylation); 2.5254 ± 0.1404 (CYP2C11, testosterone 16-α-hydroxylation); 1.0661 ± 0.0613 (CYP3A, testosterone 6-β-hydroxylation); 0.0766 ± 0.0049 (CYP3A, testosterone 2-β-hydroxylation). **B** The effect of age on CYP protein levels in the liver of senescent Dark Agouti wild-type rats (senescent WT). All values are the mean ± SEM of 8–10 rats. The representative CYP protein bands of the Western immunoblot analysis are shown. Statistical significance is shown as *p < 0.05; ***p < 0.001; ****p < 0.0001 vs. mature WT (Student’s t-test or Mann–Whitney U test, see Results). **C** The effect of age on cytochrome P450 and b_5_ mRNA expression levels in the liver of senescent Dark Agouti wild-type rats (senescent WT). Results are expressed as percent of the control (mature WT rats). All values are the mean ± SEM of 9–12 rats. Statistical significance is shown as *p < 0.05; **p < 0.01; ***p < 0.001; ****p < 0.0001 vs. mature WT (Student’s t-test or Mann–Whitney U test, see Results). b_5_, cytochrome b_5_

The activity of liver CYP1A, estimated as the rate of caffeine C-8-hydroxylation or 3-N-demethylation, decreased with aging to 50% of the control (mature WT) value, respectively, in the liver of senescent WT rats (Student’s t-test; p = 0.0001; t_24_= 4.545) and 59% (Student’s t-test; p < 0.0001; t_24_ = 6.013) (Fig. 1A). The enzyme protein level was reduced to 23% of the control (Student’s t-test; p < 0.0001; t_16_ = 10.01) (Fig. 1B), and was compatible with the decrease in the mRNA levels of CYP1A1 (MWU; p < 0.0001; U = 0; n = 12/12) and CYP1A2 (MWU; p = 0.0024; U = 13; n = 9/12) (Fig. 1C) in senescent WT rats.

The activity of CYP2A, tracked as the rate of testosterone 7α-hydroxylation and the protein level of the enzyme did not change significantly with age (Fig. 1A, B). At the same time, CYP2A2 mRNA level decreased (Student’s t-test; p = 0.0442; t_20_ = 2.147) and CYP2A1 mRNA level showed such a tendency in senescent WT rats in comparison to mature WT animals (Fig. 1C).

The CYP2B activity, tested as the rate of 16β-hydroxylation of testosterone, was diminished in the liver of senescent WT rats to 55% (Student’s t-test; p < 0.0001; t_24_ = 7.433) of the control value in mature WT rats (Fig. 1A). The decrease in the enzyme activity was accompanied by a significant decline in enzyme protein level down to 79% (Student’s t-test; p = 0.0277; t_16_ = 2.422) of that in the mature WT animals (Fig. 1B). The reduced mRNA levels of CYP2B1 (MWU; p < 0.0001; U = 0; n = 11/10), and CYP2B2 (Student’s t-test; p = 0.0212; t_18_ = 2.525) (Fig. 1C) were consistent with the observed changes of the enzyme activity and protein level.

The CYP2C6 activity, assessed as a rate of 7-hydroxylation of warfarin, decreased significantly with age to 72% (Student’s t-test; p = 0.0003; t_24_ = 4.220) of the control value in senescent WT animals (Fig. 1A). The protein level of this enzyme did not change, while the mRNA level decreased (not significantly) with age (Fig. 1B, 1C).

The activity of hepatic CYP2C11, determined as the rate of testosterone 2α- and 16α- hydroxylation, was reduced to 51% (Student’s t-test; p < 0.0001; t_24_ = 6.388) and 46% (Student’s t-test; p < 0.0001; t_24_ = 6.776), respectively, in the liver of senescent WT rats compared to mature WT rats (Fig. 1A). The enzyme protein level decreased to 43% (MWU; p = 0.001; U = 4; n = 9/8) of the control value (Fig. 1B), and was congruent with lowered (though not significantly) CYP2C11 mRNA level (Fig. 1C).

The activity of CYP3A, evaluated as the rate of testosterone 2β- or 6β-hydroxylation, decreased to 65% (Student’s t-test; p < 0.0001; t_24_ = 5.253) and 54% (Student’s t-test; p < 0.0001; t_24_ = 4.686) of the control value, respectively, in senescent WT rats (Fig. 1A). The CYP3A protein level was simultaneously reduced down to 53% of the control value (Student’s t-test; p < 0.0001; t_16_ = 6.344), compared to the control mature WT animals (Fig. 1B). The markedly lowered *CYP3A1* (MWU; p < 0.0001; U = 0; n = 12/11) and *CYP3A2* (MWU; p < 0.0010; U = 7; n = 10/9) mRNA levels (Fig. 1C) positively correlated with changes detected in the enzyme activity and protein level.

### The influence of TPH2-deficiency on the expression and activity of CYP enzymes in the liver of senescent male Dark Agouti rats (senescent TPH2-KO vs. senescent WT rats)

In the second part of our study, we concentrated on the effect of TPH2 deprivation on the CYP enzymes’ expression and activity in senescent male Dark Agouti rats. The senescent (21.5-month-old) and senescent TPH2-KO group contained 12 rats each.

**Fig. 2:**
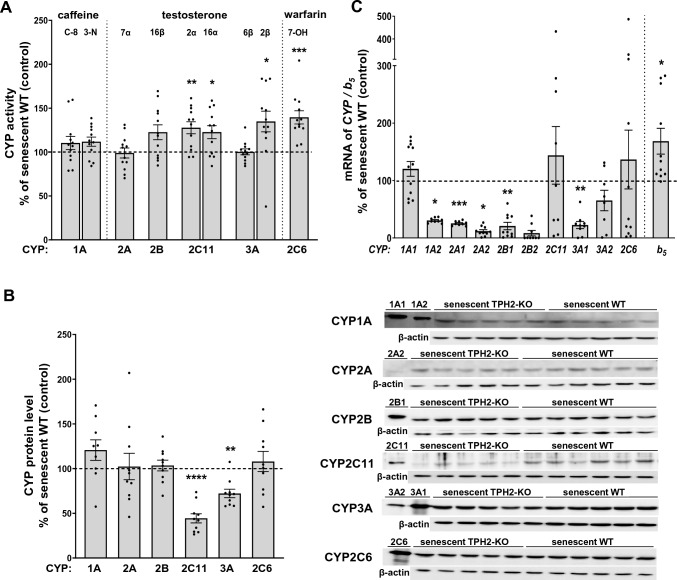
The effect of brain tryptophan hydroxylase 2 (TPH2) deficit on the cytochrome P450 enzyme activities and the expression levels of cytochrome P450 (CYP) and b_5_ genes in the liver of senescent *TPH2-KO* Dark Agouti male rats. **A** The effect of brain TPH2 deficit on the CYP enzyme activities in the liver of senescent TPH2-KO Dark Agouti male rats. All values are the mean ± SEM of 12 animals. Statistical significance is shown as *p < 0.05; **p < 0.01; ***p < 0.001 vs. senescent wild-type (senescent WT) (Student’s t-test). The control (senescent WT) values are as follows (nmol/mg of protein/min): 0.0137 ± 0.0014 (CYP1A, caffeine C-8-hydroxylation); 0.0034 ± 0.0002 (CYP1A, caffeine 3-N-demethylation); 0.4612 ± 0.0290 (CYP2A, testosterone 7-α-hydroxylation); 0.0354 ± 0.0029 (CYP2B, testosterone 16-β-hydroxylation); 0.0023 ± 0.0001 (CYP2C6, warfarin 7-hydroxylation); 1.2577 ± 0.0624 (CYP2C11, testosterone 2-α-hydroxylation); 1.5692 ± 0.0849 (CYP2C11, testosterone 16-α-hydroxylation); 0.7138 ± 0.0541 (CYP3A, testosterone 6-β-hydroxylation); 0.0474 ± 0.0031 (CYP3A, testosterone 2-β-hydroxylation). **B** The effect of brain TPH2 deficit on CYP protein levels in the liver of senescent TPH2-KO Dark Agouti male rats. All values are the mean ± SEM of 8–10 animals. The representative CYP’s protein bands of the Western immunoblot analysis are presented. Statistical significance is shown as **p < 0.01; ****p < 0.001 vs. senescent WT (Student’s t-test). **C:** The effect of brain TPH2 deficit on cytochrome P450 and b_5_ mRNA expression levels in the liver of senescent TPH2-KO Dark Agouti male rats. Results are expressed as the per cent of the control (senescent WT rats). All values are the mean ± SEM of 8–12 animals. Statistical significance is shown as *p < 0.05; **p < 0.01; ***p < 0.001 vs. senescent WT (Student’s t-test or nonparametric Mann–Whitney U test, see Results). b_5_, cytochrome b_5_

The activity and protein levels of CYP1A, CYP2A, and CYP2B were unchanged in the liver of senescent TPH2-KO rats compared to senescent male WT rats (Fig. 2A and B), though the mRNA levels of *CYP1A2* (Student’s t-test; p = 0.0189; t_1__9_ = 2.565)*, 2A1* (MWU; p = 0.0001; U = 7; n = 12/10)*, 2A2* (Student’s t-test; p = 0.0151; t_19_ = 2.672), and *CYP2B1* (Student’s t-test; p = 0.0070; t_20_ = 3.008) were significantly decreased by the mutation (Fig. 2C).

The activity of CYP2C6 increased significantly in senescent TPH2-KO rats up to 140% of the value obtained in senescent WT animals (Student’s t-test; p = 0.0003; t_22_ = 4.223), but the protein and mRNA levels of this enzyme did not change markedly in senescent TPH2-deficient rats compared to senescent WT animals (Fig. 2B, C).

The CYP2C11 activity was significantly elevated in the liver of senescent TPH2-deficient rats to 128% (testosterone 2α-hydroxylation: Student’s t-test; p = 0.0049; t_20_ = 3.161) and 123% (testosterone 16α-hydroxylation: Student’s t-test; p = 0.0270; t_20_ = 2.386) of those in senescent WT rats (Fig. 2A). In contrast, the enzyme protein level decreased to 44% of that observed in senescent WT animals (Student’s t-test; p < 0.0001; t_17_ = 5.350) (Fig. 2B). The mRNA level of *CYP2C11* showed a slight increase under TPH2-deficient conditions in senescent animals (Fig. 2C).

The activity of liver CYP3A, estimated as the rate of 6β-hydroxylation of testosterone did not change, but when assessed as the rate of 2β-hydroxylation of testosterone (a more specific CYP3A reaction in the rat) it increased in senescent TPH2-KO rats up to 135% of the value found in senescent WT rats (Student’s t-test; p = 0.017; t_22_ = 2.582) (Fig. 2A). In contrast to the enzyme activity, the levels of CYP3A protein (Student’s t-test; p = 0.0012; t_16_ = 3.946) and mRNA of *CYP3A1* (Student’s t-test; p = 0.0021; t_16_ = 3.661) were reduced (*CYP3A2 mRNA* showed such a tendency) in senescent animals with the deficit of TPH2 (Fig. 2B and 2C, respectively).

### Estimation of cytochrome b_5_ expression

In addition to the examination of cytochrome P450, the expression of cytochrome b_5_, an electron transporter from NADH to cytochrome P450 was measured. The level of cytochrome b_5_ mRNA significantly decreased with age in senescent rats (MWU; p = 0.0374; U = 32; n = 11/12) (Fig. 1C). However, under conditions of TPH2 deficiency, an increase in cytochrome b_5_ expression (mRNA) was observed in senescent TPH2-KO rats (MWU; p = 0.0336; U = 28; n = 11/11) (Fig. 2C).

### The effect of aging and TPH2-deficiency on the concentration of hormones in blood serum.

**Fig. 3:**
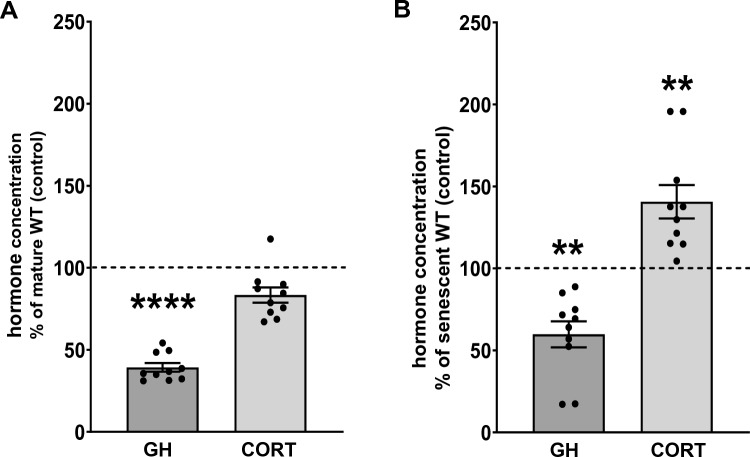
The levels of hormones in the blood serum of wild-type mature, wild-type senescent, and senescent TPH2-deficient male Dark Agouti rats. **A** The effect of age on growth hormone and corticosterone levels in blood serum of senescent wild-type Dark Agouti rats (senescent WT). All values are the mean ± SEM of 10 animals. The control values (mature WT) for GH is 792.3 ± 64 (pg/ml) and for CORT is 49.6 ± 7.7 (ng/ml). Statistical significance is shown as ****p < 0.0001 vs. mature WT (Student’s t-test). GH, growth hormone; CORT, corticosterone. **B** The effect of cerebral serotonin deficit on growth hormone and corticosterone levels in blood serum of senescent *TPH2-KO* Dark Agouti male rats. All values are the mean ± SEM of 10 animals). The control values (senescent WT) for GH is 415.8 ± 40 (pg/ml) and for CORT is 46.4 ± 4.2 (ng/ml). Statistical significance is shown as **p < 0.01 vs. senescent WT (Student’s t-test). GH, growth hormone; CORT, corticosterone

The ELISA test showed a significant decline in the serum concentration of growth hormone (Student’s t-test; p < 0.0001; t_18_ = 7.082), but we did not observe any significant changes in the level of corticosterone with aging at the time of experiment (Fig. 3A). TPH2 deficiency caused a further decrease in serum GH concentrations (Student’s t-test; p = 0.0047; t_18_ = 3.228), while the level of corticosterone increased significantly in senescent TPH2-deficient male rats compared to senescent WT rats (Student’s t-test; p = 0.0079; t_18_ = 2.985) (Fig. 3B).

## Discussion

The results of our comprehensive studies into the expression and activity of cytochrome P450 in senescent rats show that hepatic CYP enzymes function less effectively with age, which may impair drug metabolism. The decreased activities of the tested drug-metabolizing enzymes (CYP1A, 2B, 2C11, 3A and 2C6) were found. The observed decline in the hepatic CYP enzyme activities corresponded well with the diminished CYP protein and mRNA levels. It coincided with a decrease in the serum GH level, which is a positive regulator of CYP2C11 and CYP3A enzymes and contributes to the regulation of other CYP enzymes [23]. In addition, the expression of cytochrome b_5_ fell down. Cytochrome b_5_ is an electron transport protein cooperating with cytochrome P450 hemoprotein, transferring an electron from NADH over NADH-cytochrome b_5_ reductase to ferric cytochrome P450 [24]. Therefore, its decreased expression found in our experiment might contribute to the reduction in CYP enzyme activities including the diminished CYP2C6 activity which was accompanied by a non-significant change in the level of the enzyme protein [25]. Similar CYP down-regulation was observed in our previous studies for CYP2D in male and female Dark Agouti rats [13, 14].

In general, the results of our thorough studies, which measured cytochrome P450 enzymes’ activity and the levels of enzyme protein and mRNA in mature and senescent rats in male Dark Agouti rats remain in agreement with those obtained by other authors who measured the expression and/or activity of drug-metabolizing CYP enzymes in other strains of rats. In particular, similar results were obtained for CYP2C11, CYP3A2, and serum/plasma hormone levels in senescent male Wistar rats [7, 8] and male Sprague–Dawley rats [5, 11]. In fact, the decreases in CYP1A activity measured as a rate of caffeine C-8-hydroxylation and 3-N-demethylation in senescent male Sprague–Dawley rats found in our experiment are consistent with those observed using other CYP-specific metabolic reactions, such as ethoxyresorufin-O-deethylation (mainly catalyzed by CYP1A1), methoxyresorufin-O-demethylation (catalyzed by CYP1A1 and CYP1A2), and pentoxyresorufin-O-depenthylation (catalyzed by CYP1B1 and CYP1A2), which were measured in male Sprague–Dawley rats at different stages of aging [5]. However, some differences have been noticed regarding cytochrome b_5_, as no change in its mRNA was noticed by Yun and coworkers in aging male Sprague–Dawley rats [5]. The alterations in cytochrome P450 observed in rats also occur in elderly people, however, human CYP3A4 activity does not seem to practically change with aging [3, 8, 26, 27], which should be taken into consideration during pharmacotherapy in clinical practice [28].

Based on the abovementioned results obtained in our laboratory, our following studies were carried out on senescent Dark Agouti TPH2-KO male rats deprived of brain serotonin. Physiological regulation of hepatic cytochrome P450 engages pituitary, adrenal and thyroid hormones (growth hormone, corticosterone/cortisol, triiodothyronine and thyroxine), the synthesis and release of which are controlled by the brain nervous system. Our previous studies carried out after intracerebral injection of specific neurotoxins or receptor agonists showed that monoaminergic systems of the brain are engaged in the central neuroendocrine regulation of hepatic cytochrome P450 (reviewed in [15]). In the aging brain the levels of monoaminergic neurotransmitters including serotonin are reduced [29–31], which may affect the neuroendocrine regulation of different physiological processes such as CYP-mediated oxidative metabolism. On the other hand, some *TPH2* polymorphisms found in humans may affect serotonin synthesis and are associated with neuropsychiatric disorders [32–34]. To investigate the role of brain serotonin in the regulation of liver cytochrome P450 during aging, the next part of the studies was carried out on male Dark Agouti rats with knockout of brain tryptophan hydroxylase TPH2 *(TPH2-KO)*, an animal model of serotonin deficiency in the brain [13, 16]. As shown in our previous works, only trace amount of serotonin could be found in the brains of those mutated animals [14].

The results of the present studies performed on TPH2-KO rats suggest that brain serotonin regulates cytochrome P450 expression and activity in senescent animals. The deprivation of the neurotransmitter in the brain decreased the mRNA level of most of the liver CYP enzymes studied (CYP1A2, CYP2A1/2, CYP2B1/2, CYP3A1/2), lowered the protein level of CYP2C11 and CYP3A, and reduced serum GH concentration. In contrast, the activities of CYP2C11, CYP3A, and CYP2C6, and serum corticosterone concentration were increased. Moreover, the mRNA level of cytochrome b_5_ in the liver was enhanced. The observed inconsistencies between the protein level and activity of CYP2C11 and CYP3A on the one hand, and between the mRNA and protein level of CYP1A, CYP2A, CYP2B, and CYP2C11 on the other hand, suggest a complex mechanism of cytochrome P450 regulation produced by TPH2-deficiency in senescent rats, involving transcriptional regulation (decreased mRNA), as well as posttranscriptional or posttranslational modifications (e.g. changes in protein phosphorylation or serotonylation) [35–37]. In addition, alterations in other components of the mixed-function oxidase system such as an increase in cytochrome b_5_ expression may accelerate substrate oxidation and thus compensate for the decreased protein levels of CYP2C11 and CYP3A.

Interestingly, changes in cytochrome P450 expression (the levels of *CYP* mRNAs and proteins) evoked by serotonin deficit in the brain of senescent TPH2-KO Dark Agouti rats differ from those previously observed in our studies in mature WT Wistar rats (reviewed in [15]). The general damage to the brain serotonergic system via injection of a selective neurotoxin (5,7-dihydroxytryptamine) to the rostral raphe nuclei (dorsal and median raphe nuclei) projecting to the forebrain mostly leads to an opposite effect on the expression of CYP enzymes in the liver. An increase in the expression and activity of CYP1A, CYP2C11, and CYP3A accompanied by an elevation in serum GH and corticosterone concentration was observed [38]. The more detailed studies with injection of the neurotoxin or selective serotonergic receptor agonists to the hypothalamic paraventricular nuclei (PVN) or arcuate nuclei (ARC) revealed that the serotonergic innervation of PVN regulates negatively (via 5-HT_1A_ receptor and somatostatin-GH pathway) while that of ARC positively (via 5-HT_2C_ receptor and GHRH-GH pathway) the expression/activity of CYP2C11 and CYP3A enzymes [21, 39, 40]. The opposite results of the deprivation of serotonin in the brain on the neuroendocrine regulation of hepatic cytochrome P450 in mature and senescent rats (negative and positive, respectively), though observed in different experimental models suggest that, at physiological conditions, in adult rats, the negative effect of PVN serotonin predominates over the positive effect of ARC serotonin, while in senescent animals the effect of ARC serotonin prevails in the regulation of expression of liver cytochrome P450 in response to the diminished serum GH concentration in those animals (Table 1).

**Table 1:** The effect of brain serotonin deficiency on the expression of liver cytochrome P450 and serum hormone concentrations in mature and senescent male rats

Experimental model	Hormones		Cytochrome P450 activity		Cytochrome P450 protein level		Cytochrome P450 mRNA level	
	Increase	Decrease	Increase	Decrease	Increase	Decrease	Increase	Decrease
^a^Lesion of the brain serotonergic system: injection of 5,7-DHT into the raphe nuclei DRN and MRN of mature male Wistar rats	GH CORT TST	T_3_	CYP1A CYP2C11 CYP3A		CYP1A CYP2C11 CYP3A		*CYP1A1/2* *CYP2C11* *CYP3A1*	
*TPH2-KO* senescent male Dark Agouti rats	CORT	GH	CYP2C6 CYP2C11 CYP3A			CYP2C11 CYP3A		*CYP1A2* *CYP2A1/2* *CYP2B1* *CYP3A1*

The main differences between results obtained in the two experimental paradigms are underlined

*5,7-DHT* the neurotoxin 5,7-dihydroxytryptamine; *DRN* dorsal raphe nuclei; *MRN* median raphe nuclei; *TPH2* brain tryptophan hydroxylase 2; *GH* growth hormone; *CORT* corticosterone; *TST* testosterone

^a^According to Rysz et al. [38]

In conclusion, the studies carried out on male adult WT, senescent WT and senescent TPH2-KO rats indicate that aging affects the mixed function oxidase system leading to a decrease in the expression of cytochrome P450 and b_5_, and thus slowing down the rate of endogenous metabolism and drug biotransformation in the liver. The effects of TPH2-KO mutation suggest that brain serotonin is involved in the regulation of hepatic cytochrome P450 expression and activity, but the effect of brain serotonin deficiency in senescent rats seems to be different from that in mature animals. Further studies are designed to find out whether the effects of psychotropic drugs acting via the serotonergic system influence liver cytochrome P450 [41], and whether their effects differ between adults and the elderly, which is of high importance during combined pharmacotherapy [42–44].

### Supplementary Information

Below is the link to the electronic supplementary material.

Supplementary file1 (PDF 1684 KB)

## Data Availability

The datasets are available from the corresponding author upon reasonable request. All immunoblots generated during are included in Supplementary Materials.

## Abbreviations

b_5_: Cytochrome b_5_
CYP: Cytochrome P450
HPLC: High performance liquid chromatography
NADP: Nicotinamide adenine dinucleotide phosphate
NADPH: Reduced form of nicotinamide adenine dinucleotide phosphate
MWU: Mann–Whitney U test
TPH2: Tryptophan hydroxylase 2
TPH2-KO: TPH2-knockout
WT: Wild type
