# Supplementary material for: The effect of brain serotonin deficit (TPH2-KO) on the expression and activity of liver cytochrome P450 enzymes in aging male Dark Agouti rats
Source: Pharmacol Rep. 2023 Oct 17;75(6):1522–32. doi: 10.1007/s43440-023-00540-x (PMC10661807; doi:10.1007/s43440-023-00540-x)

CYPs_mature WT_senescent WT rats:

CYP1A

CYP1A_membrane 1_Fig. 1B

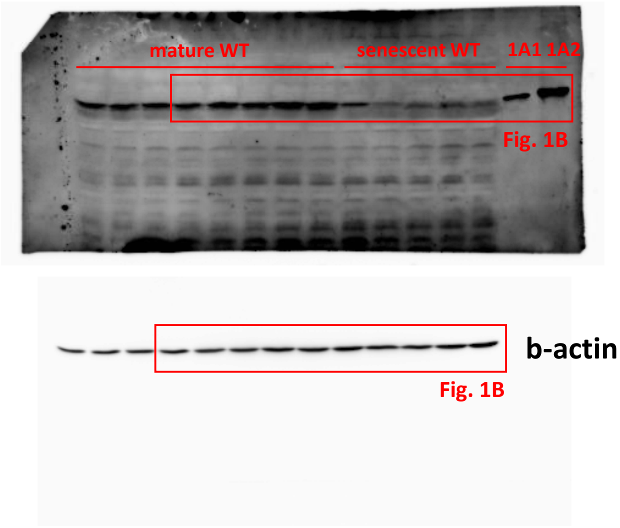

CYP1A_membrane 2

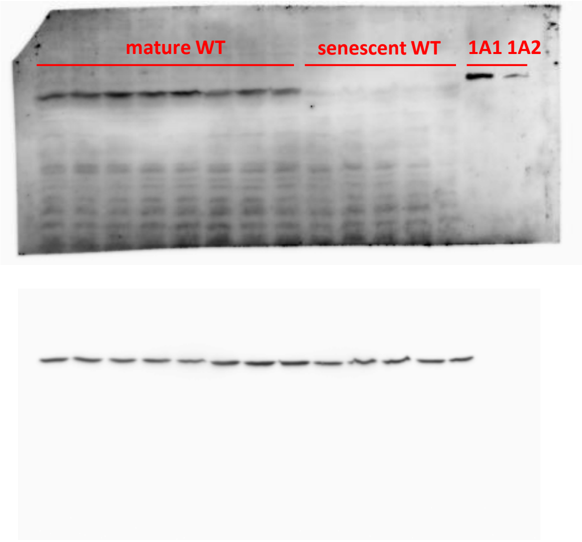

CYP2A

CYP2A_membrane 1_Fig. 1B

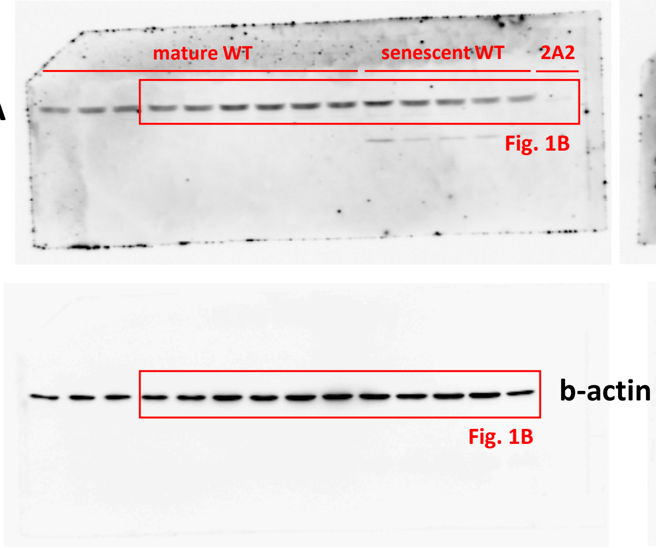

CYP2A_ membrane 2

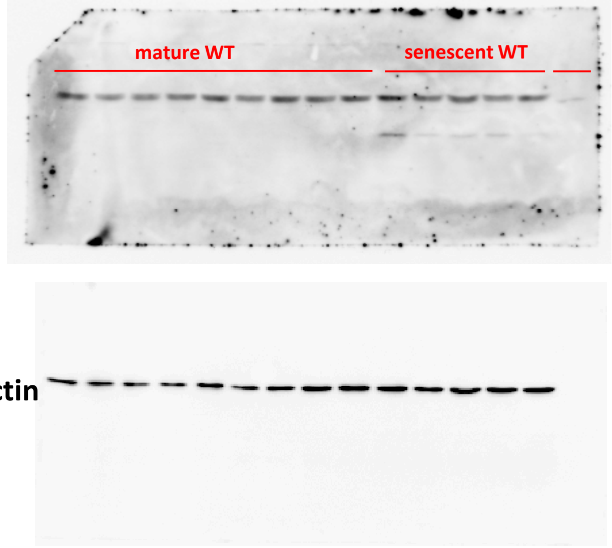

CYP2B

CYP2B_membrane 1_Fig. 1B

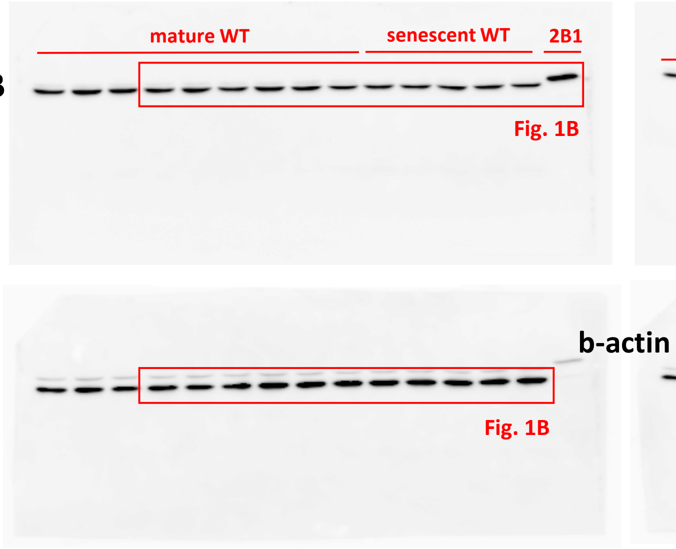

CYP2B_membrane 2

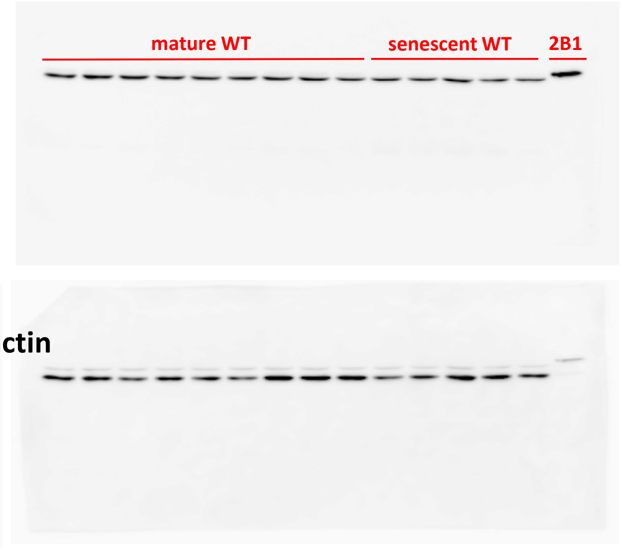

CYPs_mature WT_senescent WT rats:

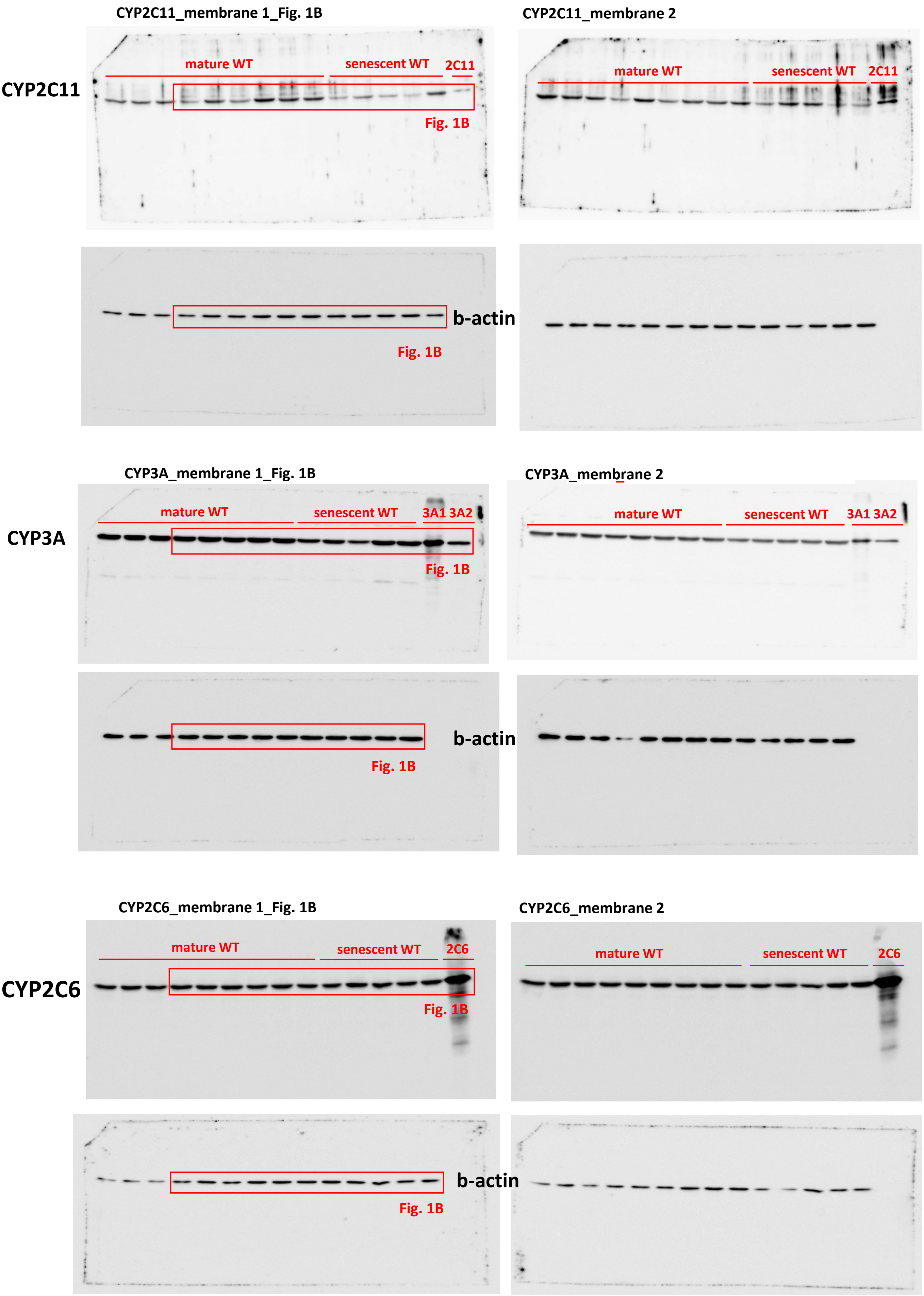

CYPs_senescent TPH2-KO_senescent WT rats:

CYP1A_membrane 1_Fig. 2B

CYP1A_membrane 2

CYP1A

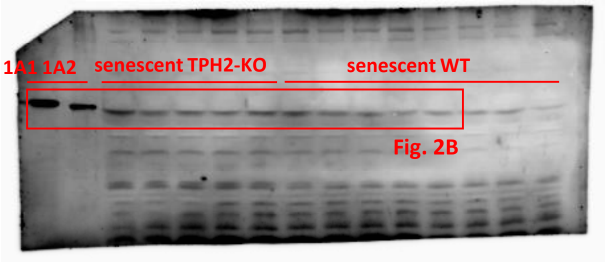

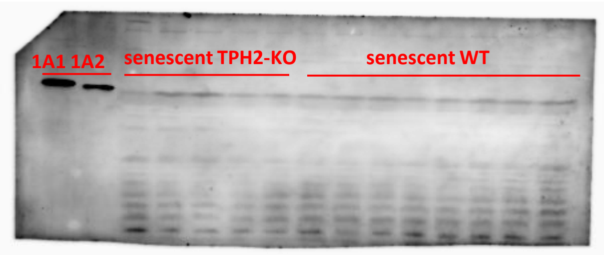

b-actin

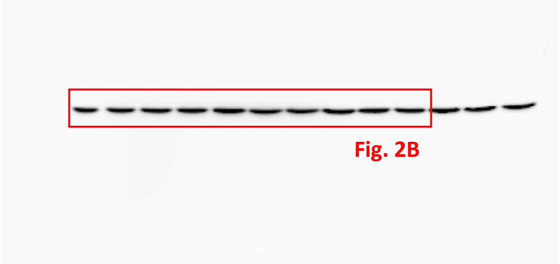

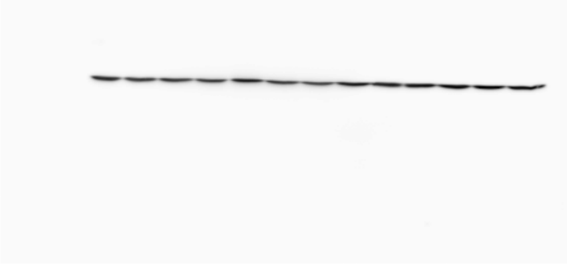

CYP2A_membrane 1_Fig. 2B

CYP2A_ membrane 2

CYP2A

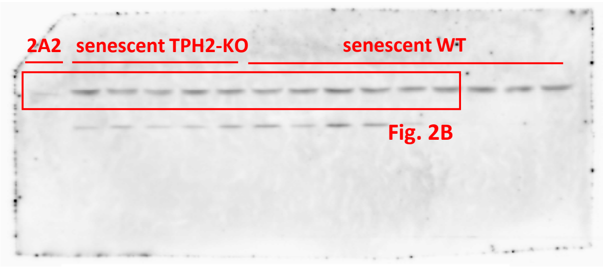

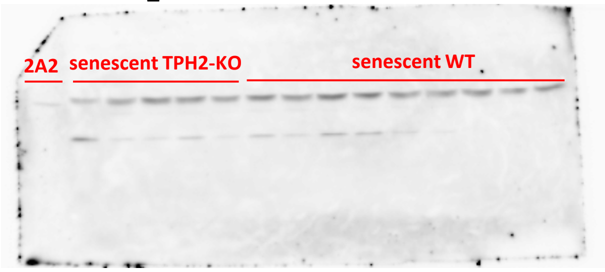

b-actin

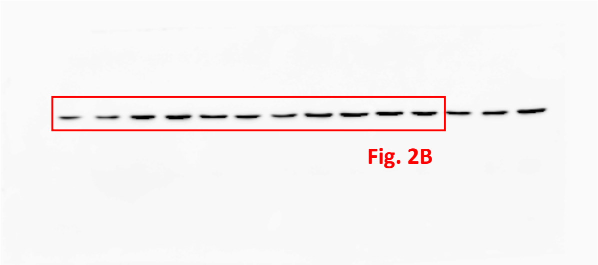

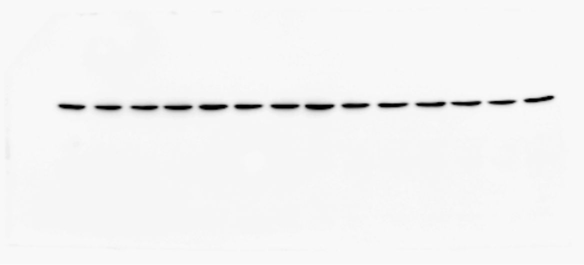

CYP2B_membrane 1_Fig. 2B

CYP2B_membrane 2

CYP2B

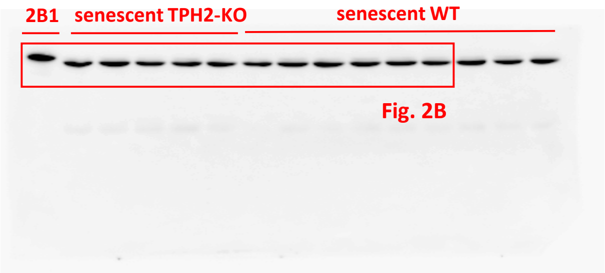

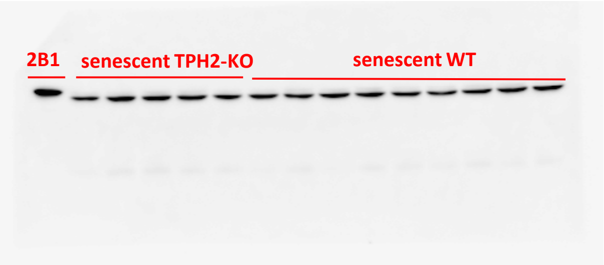

b-actin

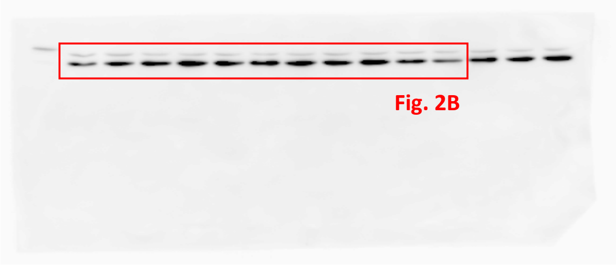

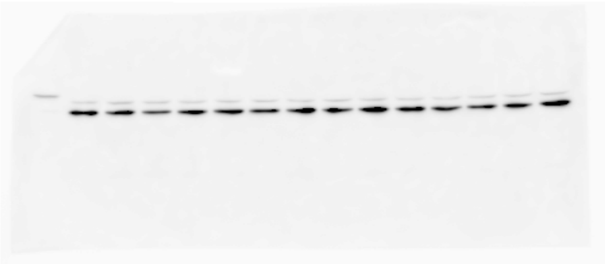

CYPs_senescent TPH2-KO_senescent WT rats:

CYP2C11_membrane 1_Fig. 2B

CYP2C11_membrane 2

CYP2C11

b-actin

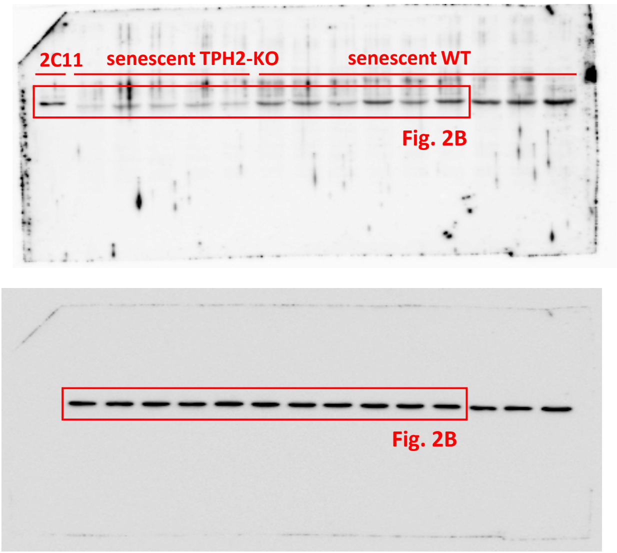

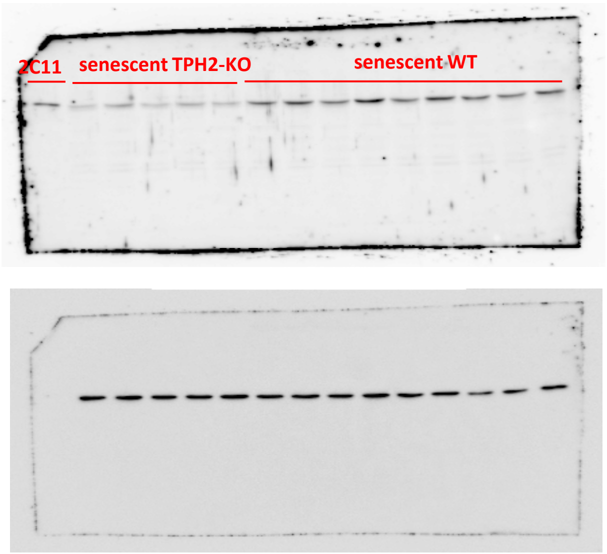

CYP3A_membrane 1_Fig. 2B

CYP3A_ membrane 2

CYP3A

b-actin

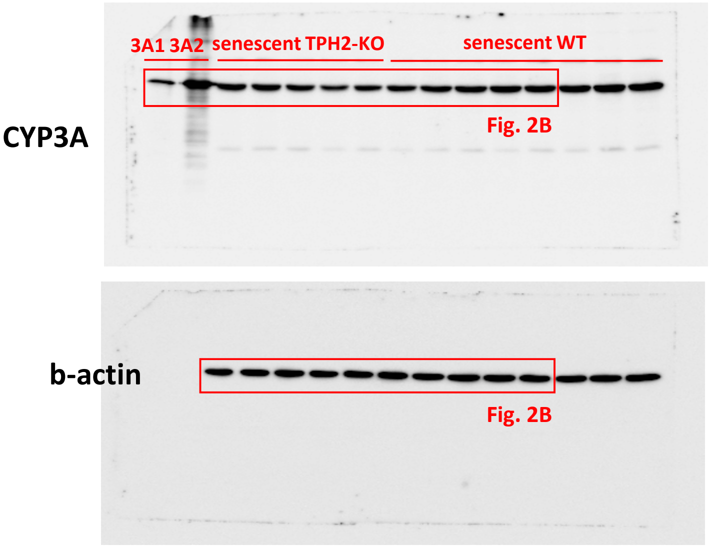

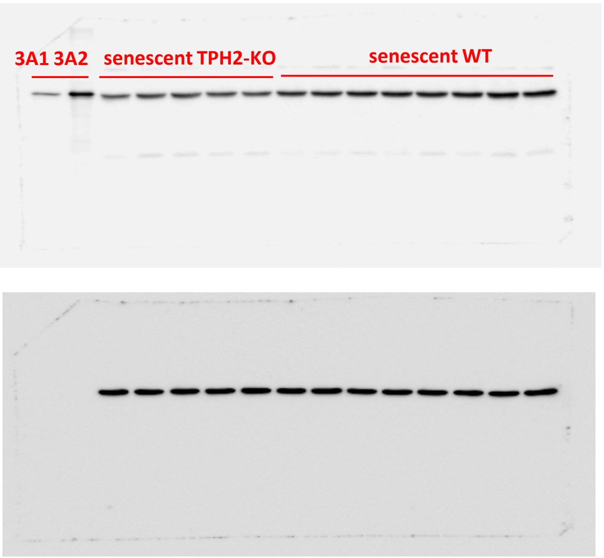

CYP2C6_membrane 1_Fig. 2B

CYP2C6_membrane 2

CYP2C6

b-actin

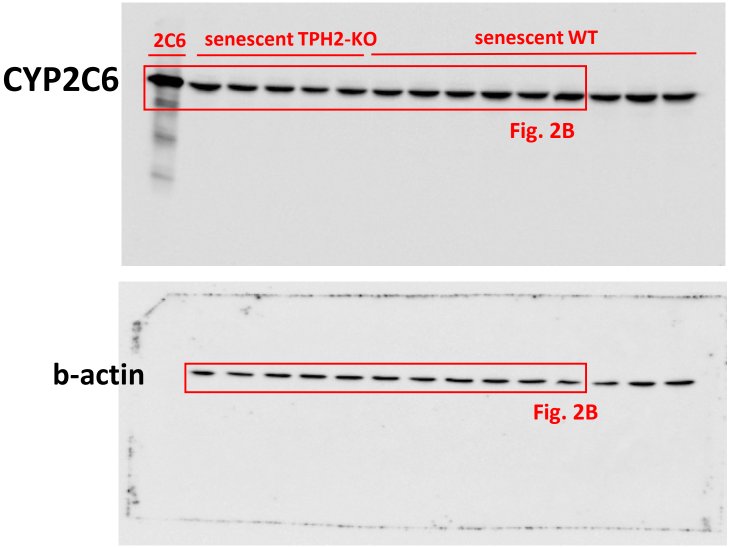

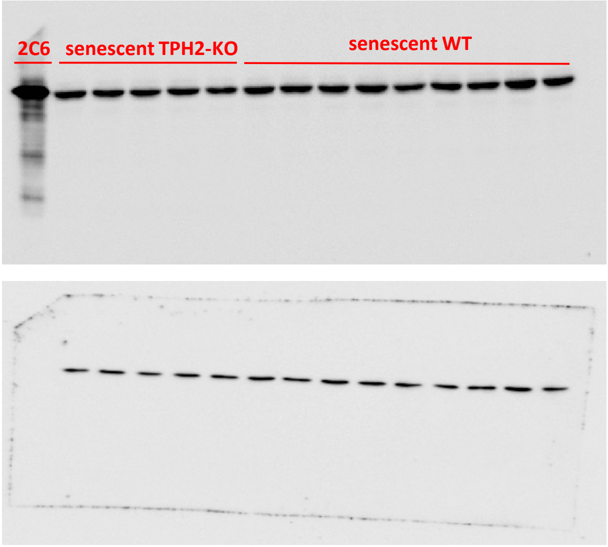

Supplement: Supplementary file 1 — Supplementary file1 (PDF 1684 KB) [file 43440_2023_540_MOESM1_ESM.pdf]
